# Characterizing CD38 expression in terminally differentiated B cells using variable lymphocyte receptor B tetramers

**DOI:** 10.3389/fimmu.2024.1451232

**Published:** 2024-10-30

**Authors:** Arundhati G. Nair, Matilde Leon-Ponte, Vy HD Kim, Gordon Sussman, Götz R.A. Ehrhardt, Eyal Grunebaum

**Affiliations:** ^1^ Developmental and Stem Cell Biology Program, Hospital for Sick Children, Toronto, ON, Canada; ^2^ Institute of Medical Sciences, University of Toronto, Toronto, ON, Canada; ^3^ Division of Immunology and Allergy, Department of Pediatrics, The Hospital for Sick Children, Toronto, ON, Canada; ^4^ Division of Clinical Immunology and Allergy, Department of Medicine, University of Toronto, Toronto, ON, Canada; ^5^ Department of Immunology, University of Toronto, Toronto, ON, Canada

**Keywords:** plasmablasts, transitional B cells, sea lamprey, antibody deficiencies, allergy, CD38, variable lymphocyte receptor

## Abstract

**Introduction:**

CD38 is an ectoenzyme receptor found on hematopoietic cells and its expression is used in the flow cytometric analysis of sub-populations of circulating B cells among peripheral blood mononuclear cells (PBMC) to aid in diagnosing patients with different antibody production defects (AbD). Monoclonal antibodies derived from the sea lamprey Variable Lymphocyte Receptor B (VLRB) are emerging as an alternative to conventional mammalian antibodies. We hypothesized that VLRB MM3 (V-CD38) which specifically recognizes CD38 in a manner correlating with its enzymatic activity could identify terminally differentiated B cells in human PBMC. Here we investigate the ability of V-CD38 as a tool to diagnose patients with diverse immune abnormalities including AbD.

**Methods:**

The expression of CD38 on CD3^-^CD19^+^CD27^+^ plasmablasts and CD3^-^CD19^+^IgM^hi^CD27^-^ transitional B cells in PBMC was analyzed by flow cytometry using V-CD38 and compared with a commercial conventional antibody to CD38 (C-CD38).

**Results:**

A highly significant correlation (p<0.001, r=0.99) between the percentages of plasmablasts recognized by V-CD38 and C-CD38 was observed among 36 healthy controls (HC), 7 patients with AbD and 24 allergic individuals (AI). The use of V-CD38 enabled improved gating of the CD38 expressing cells (CD38+), aiding in the observation that patients with AbD had significantly lower (p=0.002) CD38+ plasmablasts (0.13%±0.13%) than HC (0.52%±0.57%). Only 61.3% of the transitional B cells detected by C-CD38 were also recognized by V-CD38 (r=0.95, p<0.001) among the 67 participants. AI had significantly reduced V-CD38 and C-CD38 transitional cells compared to HC (p=0.026 and p=0.012, respectively).

**Conclusions:**

V-CD38 is a novel reagent that can assess B cells in human PBMC.

## 1 Introduction

Human CD38 is a 300 amino acid receptor and ectoenzyme that catalyzes the metabolism of cyclic ADP-ribose and nicotinic acid adenine dinucleotide phosphate (1). CD38 is expressed on the surface and in intracellular organelles of many cells of hematopoietic and non-hematopoietic origin, including NK, activated T and B lymphocytes (2). CD38 has diverse roles in immune responses, autoimmunity, and inflammation, partly through its effects on cell adhesion and signal transduction (3). CD38 is also found on plasma cells and is highly expressed on multiple myeloma cells; therefore it is targeted by several therapeutic monoclonal antibodies (mAbs), such as daratumumab and isatuximab (4).

Perturbations in the percentages of subpopulations of B cells among peripheral blood mononuclear cells (PBMC) can assist in diagnosing and managing patients with different antibody production defects (AbD) (5). Therefore, determining the expression of CD38 (CD38^+^), together with CD19, CD27, IgM and IgD, has been incorporated in the flow cytometry analysis of individuals with acquired or inborn errors of immunity. For example, reduced numbers of memory B cells are included in the classification of Common Variable Immunodeficiency (CVID), which is characterized by recurrent infections, decreased IgA or IgM and hypogammaglobulinemia (6), as not all those with reduced IgG suffer from CVID and not all patients with CVID have low IgG. Among human circulating CD19^+^ B cells, CD38 expression is low or absent on naïve (IgD^+^CD27^−^CD38^-^), marginal zone equivalent cells (IgD^+^CD27^+^CD38^-^) and switched memory B cells (IgD^−^CD27^+^CD38^-^), intermediate on transitional cells (IgM^hi^CD27^-^CD38^+/++^), and high on antibody-secreting plasmablasts (IgM^−^CD27^+^CD38^++/+++^) (7).

Antibodies to human CD38 have been generated in several mammalian species; however, the specificity and repertoire of these antibodies are restricted by *in vivo* selection mechanisms and *ex vivo* production technologies. Indeed, flow cytometry analysis of cells expressing CD38 with currently available commercial antibodies to CD38 can be challenging, with different CD38 clones and fluorescent conjugates resulting in variable intensities, expression and percentages of CD38 cells in humans. For example, one study using the anti-CD38 HIT2 clone reported that the normal range of plasmablasts (5^th^ -95^th^ percentiles) in healthy adults was 0.4% - 3.6% (8), while another study using the HB7 clone reported a range of 0% - 0.4% (9). Similarly, plasma cells, which are also identified by the expression of CD38, are present at less than 100 cells/ul in the peripheral blood of healthy individuals (10).

In contrast to mammals, jawless vertebrates, the only extant members are lampreys and hagfish, recognize antigens by using variable lymphocyte receptors (VLRs), a somatically diversifying anticipatory receptor system based on the leucine-rich repeat (LRR) as a basic structural unit, with VLR type B (VLRB) being secreted from lymphocyte-like cells similar to classical antibodies (11, 12). VLRB antibodies are stable, have high avidity and recognize their target antigen with high levels of specificity, exceeding those observed for conventional antibodies (13). For this reason, there is growing interest in employing VLRB antibodies for biomedical research and clinical practice, such as the VLRB B7 that recognizes the SARS-CoV-2 spike protein (14). Monoclonal VLRB MM3 was isolated from sea lamprey larvae immunized with bone marrow aspirate from a patient diagnosed with multiple myeloma. VLRB MM3 binds CD38 in a manner that correlates with enzymatic activity and specifically recognizes CD38 on human plasma cells from the tonsil, spleen, and bone marrow, as well as peripheral blood plasmablasts following influenza vaccination (15). To facilitate the versatile use of VLRB MM3 antibodies in combination with conventional antibody panels including conventional antibodies targeting CD38, we generated tetramers of biotinylated monomeric VLRB MM3 antibodies to CD38 (V-CD38), thereby taking advantage of the improved ability of V-CD38 to detect plasma cells in tonsil tissues (16).

We hypothesized that V-CD38 could improve the detection of CD38-expressing transitional B cells and plasmablasts in human PBMC. Therefore, the objectives of our study were to titrate V-CD38 and demonstrate that V-CD38 could recognize immunoglobulin G (IgG)-secreting plasmablasts in PBMC of healthy controls (HC), similar to a commercial monoclonal antibody against CD38 (C-CD38). Moreover, we aimed to compare the utility of V-CD38 with that of C-CD38 for the identification of plasmablasts in PBMC isolated from HC, patients with diverse antibody deficiency (AbD) and allergic individuals (AI) and analyze V-CD38’s ability to characterize B cell sub-populations.

## 2 Materials and methods

### 2.1 Patients and healthy controls

The study was conducted between September 1^st^, 2021, and May 31^st^, 2023, at the Hospital for Sick Children, Toronto and a community allergy clinic in Toronto, Ontario. The study included patients diagnosed with AbD using the European Society for Immunodeficiency (ESID) criteria (6), and AI diagnosed by an experienced allergist (GS). AI enrollment criteria included diverse atopic conditions such as food, drug and environmental allergies, asthma, allergic rhinitis, urticaria and anaphylaxis, diagnosed and managed following national and international professional society guidelines. AI were excluded if they suffered from an immunodeficiency, or severe infection, or were receiving immunoglobulin replacement, or immune suppressive/modulatory medications, which may interfere with immune cell subsets. Patients were excluded if there was evidence for secondary cause for hypogammaglobulinemia such as protein loss, receiving immunosuppressive treatments, having active, latent, or inadequately treated infection, profound anemia (<7 g/dL), thrombocytopenia or any indication of bone marrow failure disorder. Patients were also excluded if they were suffering from malignancy or had a history of HIV infection or myelodysplasia. Healthy controls (HC) included volunteers with no clinical or laboratory evidence for abnormal antibody production, or allergies. The study was approved by the Research Ethics Board at the Hospital for Sick Children, Toronto (REB 1000079651) and samples were collected after obtaining written consent from participants or their guardians in accordance with as required by the Declaration of Helsinki.

### 2.2 Peripheral mononuclear blood cell collection and storage

PBMC were isolated from blood using SepMate PBMC Isolation Tubes (STEMCELL Technologies, Vancouver BC, Canada). PBMC were frozen in 90% RPMI media (80% RPMI, 20% FBS, and Pen-Strep; Wisent, St. Bruno QC, Canada) and 10% DMSO (Sigma Life Science, Burlington, MA, USA) and stored at -80°C before thawing and analysis by flow cytometry.

### 2.3 Biotinylated VLRB tetramer generation and analysis of antibodies

Biotinylated VLRB MM3 and VLRB B7 antibodies were generated as described previously (16). To tetramerize the biotinylated monomers, a 1:60 dilution of streptavidin-PE (SouthernBiotech, Birmingham, AL, USA) in FACS staining buffer (Invitrogen, Carlsbad, CA, USA) was created, and 47.5 uL of this dilution was added to 2.5 uL of biotinylated monomeric VLRB reagent stepwise in 10 uL increments. A 10-minute incubation time was allowed after each increment. Titration of antibodies was performed by serial dilutions of biotinylated VLRB MM3, and VLRB B7 (negative control against SARS-CoV-2 spike protein) on the CD38+ Burkitt’s lymphoma-derived Daudi cell line (ATCC, Manassas, VA, USA). The antibody dilution that provided the highest stain index was selected for further experiments.

### 2.4 Flow cytometry

After the thawing of the PBMC, single-cell suspensions were incubated with “B cell mAbs mix” and V-CD38-PE tetramers in the dark for 20 minutes. The B cell mAbs mix included anti-CD3 Brilliant Ultraviolet (BUV) 395, anti-CD19 Brilliant Violet (BV) 421, anti-CD27 phycoerythrin-cyanine 7 (PE-Cy 7), anti-IgM fluorescein isothiocyanate (FITC), anti-IgD BUV 737, anti-CD21 allophycocyanin (APC) and the Fixable Viability Stain (FVS) 575V (all from BD Biosciences, San Jose, CA, USA). The gating strategy was adapted from Warnatz and Schlesier (8) with CD27^hi^CD38^hi^ plasmablasts gating following the Euroflow guidelines (5). In some experiments, VLRB B7 tetramers were used as a negative control.

To further confirm that V-CD38 was recognizing plasmablasts, cells were assessed for the presence of intracellular IgG. Cells were stained as described above except for anti-CD21, fixed with IC Fixation Buffer and permeabilized with Permeabilization Buffer (both from Invitrogen, Carlsbad, CA, USA). Anti-IgG APC (BD Biosciences) was then added.

All flow cytometry experiments were acquired on a 5-laser FACS Symphony A3 Cell Analyzer (BD Biosciences) and analyzed using FlowJo software (BD Bioscience, Ashland, OR, USA). Fluorescence minus one (FMO) was established for all antibodies. In all experiments, a minimum of 2X10^5^ events were gated, allowing confident detection of populations of cells at a frequency of as low as 0.1%. Indeed, the minimum number of events assessed was in line with similar reports of 1-5 X10^5^ cells gated (17, 18), particularly when considering that some of our patients were children where blood volumes for research purposes are limited.

### 2.5 Statistical analysis

Statistical analysis was performed using GraphPad Prism v9.0.2. Brown-Forsythe and Welch ANOVA tests were used to analyze differences between patients, AI, and HC. Paired t-tests were used to investigate differences between the proportions of plasmablasts and transitional B cells identified by C-CD38 and V-CD38. Correlations were calculated using Pearson’s coefficient calculation. Results are presented as Mean ± Standard Deviation (SD). P-values <0.05 were considered statistically significant.

## 3 Results

### 3.1 Cohort characteristics

The study included 36 HC, 10 patients with AbD including 5 who were receiving immunoglobulin replacement (**Supplementary Table 1**) and 24 AI. The etiologies of the AbD were diverse and often without a known genetic etiology. Among the identified gene defects, some conditions such as BTK deficiency resulted in complete or near complete absence of CD19+ B cells and immunoglobulin production. In other patients, such as those with abnormalities in LRBA protein (19), in *STAT3-*related HyperIgE syndrome associated with normal immunoglobulin yet abnormal B cell development (20) and variable production of specific antibodies (21), in Ataxia-Telangiectasia mutated (22) and ZTTK syndrome caused by SON haploinsufficiency (23), the number of CD19+ B cells and immunoglobulin concentrations ranged from markedly reduced to normal. The HC and AI did not undergo detailed immunological evaluations, therefore there is no information about their IgG/IgA/IgM or B cell numbers. The AbD group had a male sex predominance, and their age was not significantly different than the HC (**Table 1**). In contrast, AI were predominantly female and were significantly older than the HC.

**Table 1:** Characteristics of the healthy controls, patients with antibody deficiencies and allergic individuals.

	Healthy controls	Antibody deficiencies	Allergic individuals
Total	36	10	24
Mean Age years ± SD	21.3 ± 20.4	15.8 ± 5.8, *p=0.300	45.3 ± 18.3, *p<0.001
Sex (male/female)	21/15	9/1, *p=0.045	6/18, *p=0.018.

*P values compared to healthy controls.

### 3.2 V-CD38 recognizes human CD38 on Daudi cells, peripheral blood cells and IgG-producing plasmablasts similar to C-CD38

To confirm the ability of biotinylated V-CD38 to recognize human CD38, the binding of the antibody was first tested and titrated using Daudi cells known to express CD38 (15). V-CD38 detected Daudi cells with an inverse correlation between antibody dilution and fluorescence index (**Supplementary Figure 1A**). In contrast, staining with VLRB B7, used as a negative control, did not result in any detectable fluorescence signals (**Supplementary Figure 1B**).

To date, there are no published reports on V-CD38 identifying plasmablasts in the PBMC of HC. Therefore, in preliminary studies, following the gating strategy proposed by the EuroFlow (5) we used PBMC of three HC to confirm that V-CD38 recognized human CD38^hi^CD27^hi^ plasmablasts (**Supplementary Figure 2A**) similar to C-CD38 (**Supplementary Figure 2B**). We also confirmed that freezing and thawing did not significantly alter the viability of the cells or the expression of B cell markers including the recognition of CD38 expression by V-CD38 and C-CD38 (**Supplementary Figure 3**).

**Figure 1:**
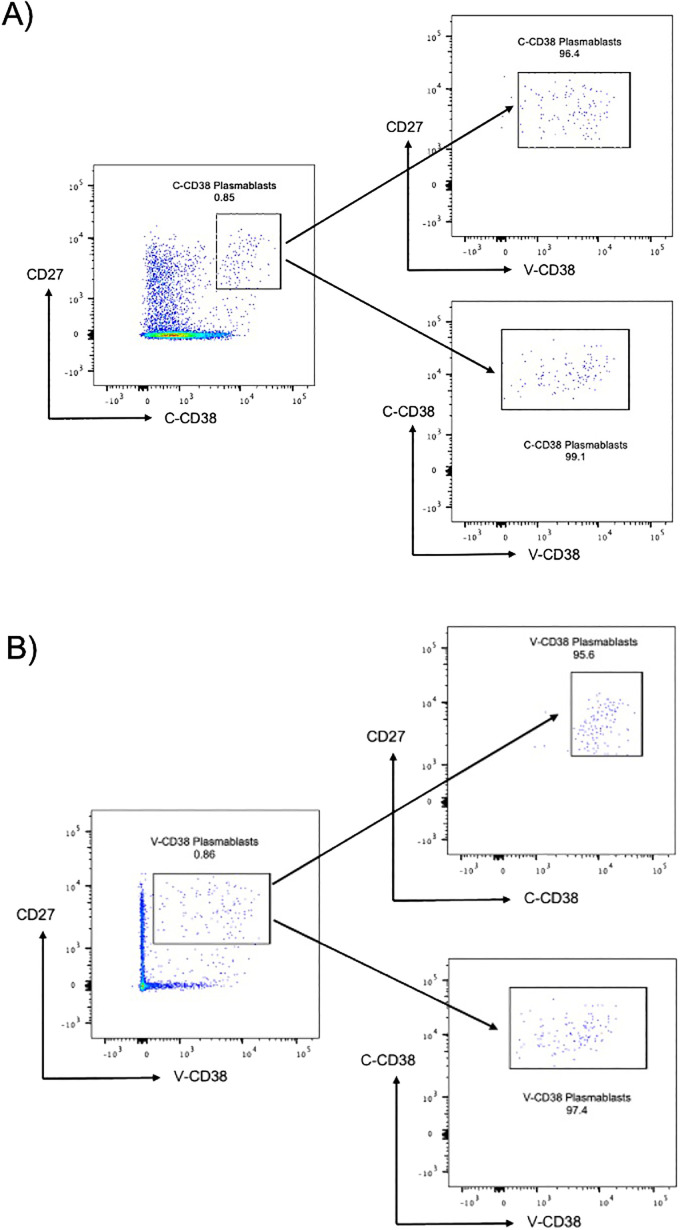
C-CD38 and V-CD38 binding to plasmablasts in peripheral blood. **(A)**. Plasmablasts were characterized as single, viable, CD3^-^CD19^+^CD27^hi^ C-CD38^hi^ cells. The arrows point to the C-CD38 plasmablasts identified by V-CD38. **(B)**. Plasmablasts were characterized as single, viable, CD3^-^CD19^+^CD27^hi^V-CD38^+^ cells. The arrows point to the V-CD38 plasmablasts identified by C-CD38.

Since plasmablasts are characterized by their ability to produce immunoglobulins, most commonly IgG, we confirmed the presence of intracellular IgG within cells recognized by CD38. Testing of the PBMC from 3 HC with V-CD38 (**Supplementary Figure 4A**) or with C-CD38 (**Supplementary Figure 4B**) demonstrated practically identical percentages of CD38^+^ IgG-producing cells (**Supplementary Figure 4C**). Indeed, 24-52% of IgG-expressing plasmablasts identified with V-CD38 or C-CD38 which is in the range expected from previous reports (24). T cells, that do not produce IgG, were used as a negative control. Furthermore, to assess the reproducibility of V-CD38, 5 replicates of an HC were assessed showing minimal standard deviations in the percentages of C-CD38 transitional B cells (0.77%±0.027%), V-CD38 transitional B cells (0.39%±0.011%), C-CD38 plasmablasts (0.76%±0.047%) and V-CD38 plasmablasts (0.76%±0.057%). Finally, taking advantage of the different binding sites of V-CD38 compared to C-CD38 (15), the two antibodies were used together. There were no differences in the percentages of gated plasmablasts whether cells were first gated on C-CD38 and then on V-CD38 (**Figure 1A**) or gating was first on V-CD38 followed by gating on C-CD38 (**Figure 1B**) demonstrating that indeed both recognized the same cells. In contrast, there is no staining with the negative control VLRB B7 antibody (data not shown).

### 3.3 V-CD38 accurately recognizes plasmablasts in healthy controls, patients with antibody deficiencies and allergic individuals

**Figure 2:**
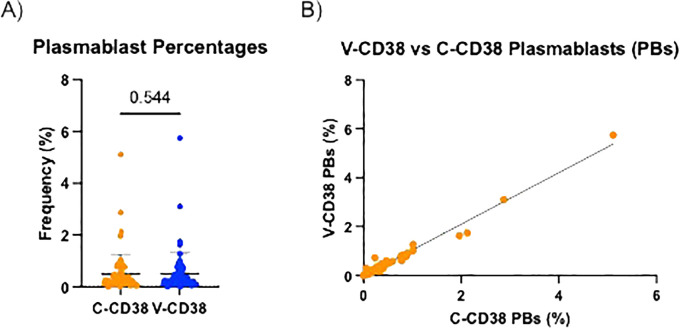
Percentages of V-CD38+ and C-CD38+ plasmablasts in the peripheral blood of healthy controls and patients. **(A)** Percentages of CD3^-^CD19^+^CD27^hi^ plasmablasts detected by C-CD38 or V-CD38 in PBMC of HC, AbD patients and AI (n=67). Statistical significance was assessed using a paired t-test. Error bars represent standard deviation. **(B)** Correlation between the percentages of CD3^-^CD19^+^CD27^hi^ plasmablasts detected with C-CD38 or V-CD38 in PBMC of HC, AbD patients and AI (n=67). Statistical significance was assessed by Pearson’s Correlation Coefficient (r=0.97, p<.001).

After establishing that plasmablasts from PBMC can be recognized similarly by V-CD38 and C-CD38, the ability of the two antibodies to identify plasmablasts was compared between a large group of HC, patients with AbD and AI. These 3 groups were chosen as they were expected to provide a wide spectrum of values. The frequencies of plasmablasts identified with V-CD38 (0.49%±0.76%) were similar (p=0.54) to the percentages identified by C-CD38 (0.50%±0.81%), with a strong positive correlation (r=0.95, p<.001) between the percentages of cells detected by the two antibodies (**Figures 2A, B**).

### 3.4 Patients with antibody deficiencies have reduced percentages of plasmablasts

**Figure 3:**
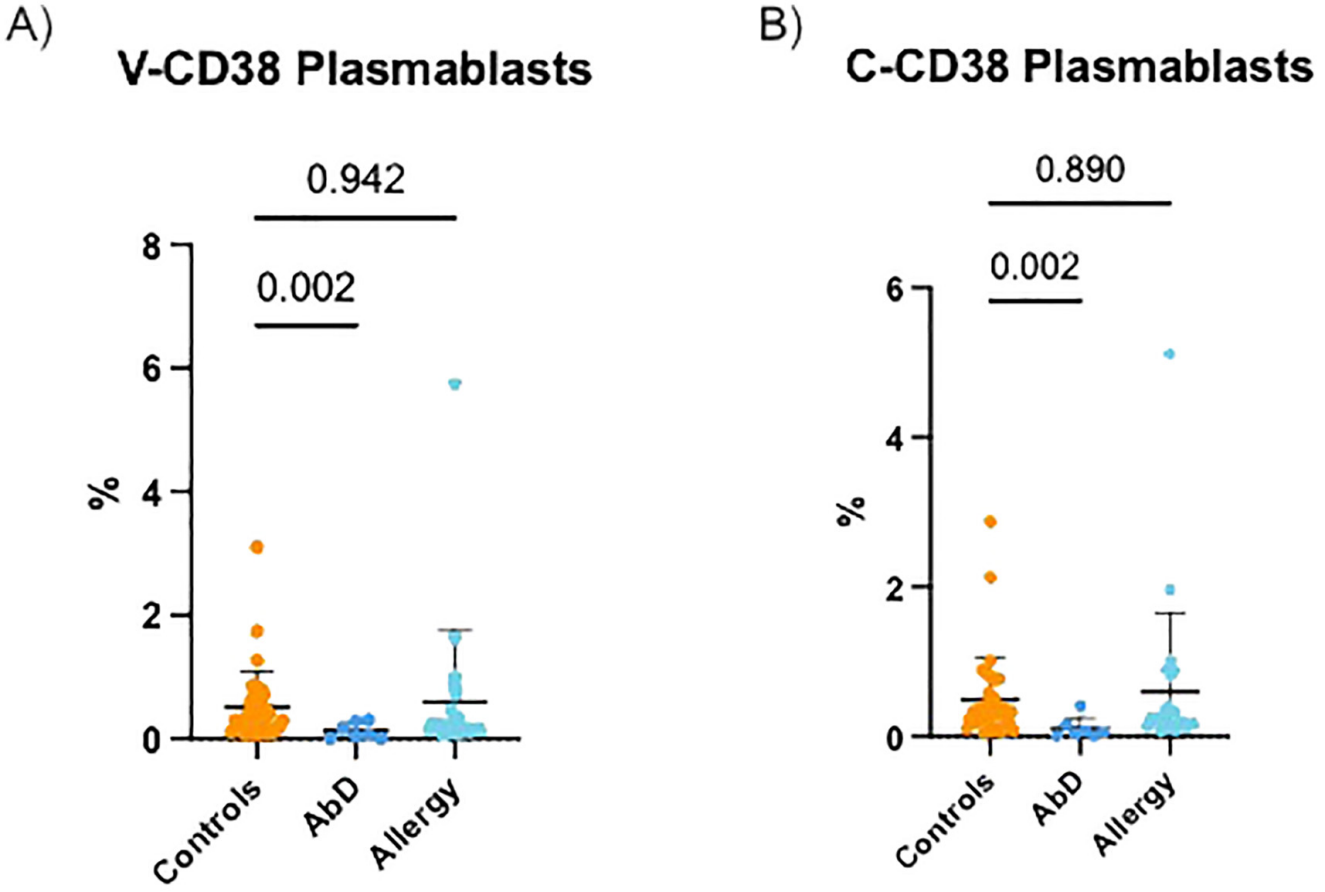
V-CD38+ and C-CD38+ plasmablasts in the peripheral blood of healthy controls, patients with diverse antibody deficiencies and allergic individuals. **(A)** Percentages of CD3^-^CD19^+^CD27^hi^ plasmablasts identified by V-CD38 among PBMC from HC (n=36), patients with AbD (n=7) and AI (n=24). **(B)** Percentages of CD3^-^CD19^+^CD27^hi^ plasmablasts identified by C-CD38 among PBMC from HC (n=36), patients with AbD (n=7) and AI (n=24).

Diverse AbD can be characterized by reduced percentages of plasmablasts (8). Therefore, the ability of V-CD38 and C-CD38 to distinguish between 36 HC, 7 patients with AbD (3 patients were excluded from analysis due to lack of CD19+ B cells) and 24 AI were compared. As demonstrated in **Figure 3A**, patients with AbD had significantly lower (p=0.002) circulating plasmablasts recognized by V-CD38 (0.13%±0.13%) than HC (0.52%±0.57%), a difference similar to that observed when C-CD38 (0.11%±0.14% vs 0.50%±0.57%) was used to identify plasmablasts (**Figure 3B**). In contrast, there was no significant difference in the percentages of plasmablasts recognized by V-CD38+ (0.59%±1.16%) or C-CD38 (0.60%±1.05%) in AI compared to HC. However, staining with V-CD38 allowed a more accurate determination of the reduced plasmablasts frequencies in patients with AbD compared to C-CD38. Indeed, using a value of 0.35% plasmablasts identified with V-CD38 as the lower limit of normal provided 100% sensitivity in identifying patients with AbD and 50% specificity, which was an improvement to the 44% specificity obtained when using C-CD38 (**Supplementary Table 2**).

### 3.5 Differences between V-CD38 and C-CD38 binding to transitional B cells

**Figure 4:**
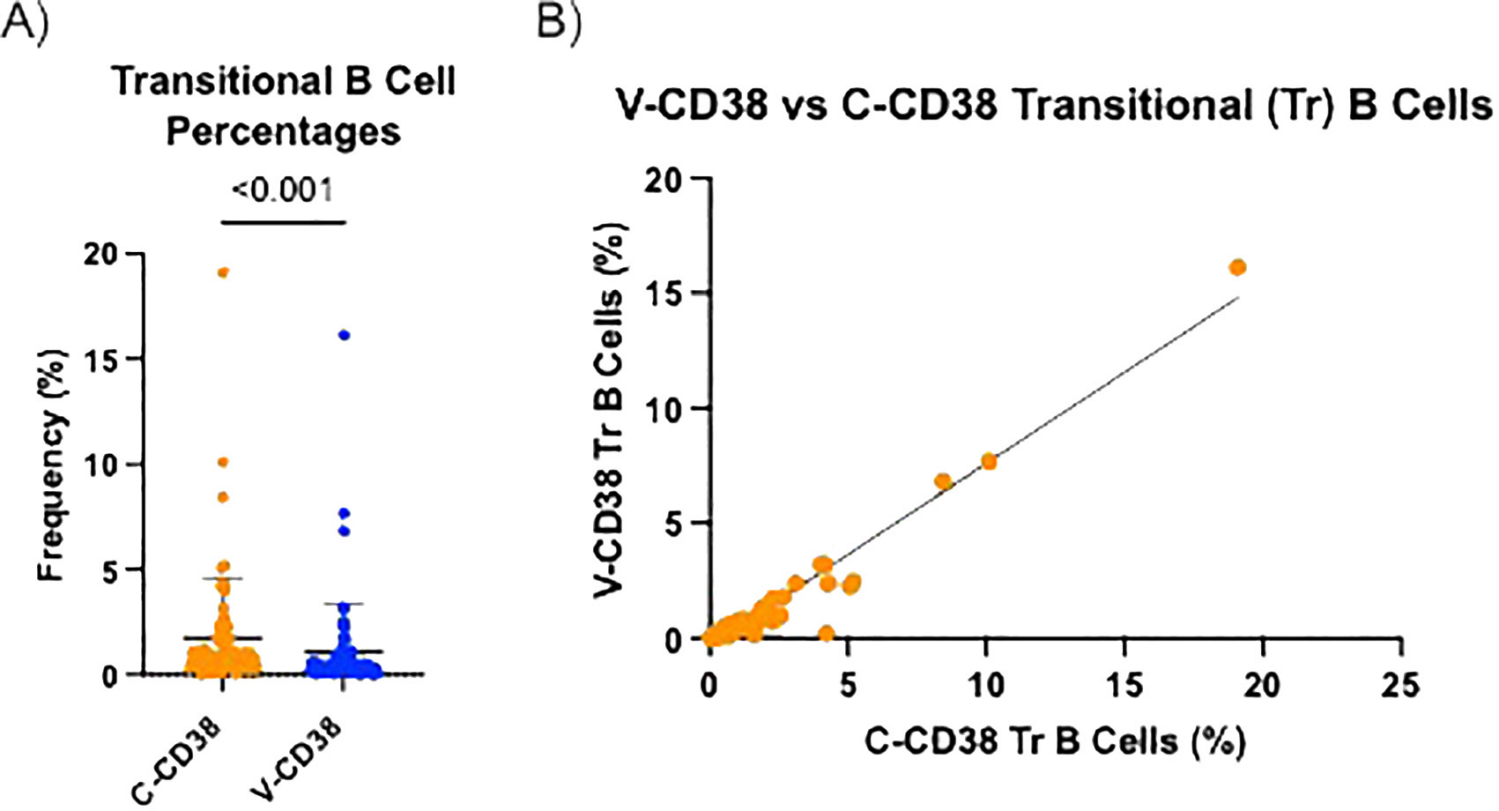
V-CD38 recognizes only some of C-CD38+ transitional B cells across all participants. **(A)** Percentage of transitional B cells recognized by C-CD38 and identified by V-CD38 (n=67). Statistical significance was assessed by paired t-test. Error bars represent SD. **(B)** Correlation between the percentages of transitional B cells recognized by C-CD38 and V-CD38 (n=67). Statistical significance was assessed by Pearson’s Correlation Coefficient (r=0.88).

**Figure 5:**
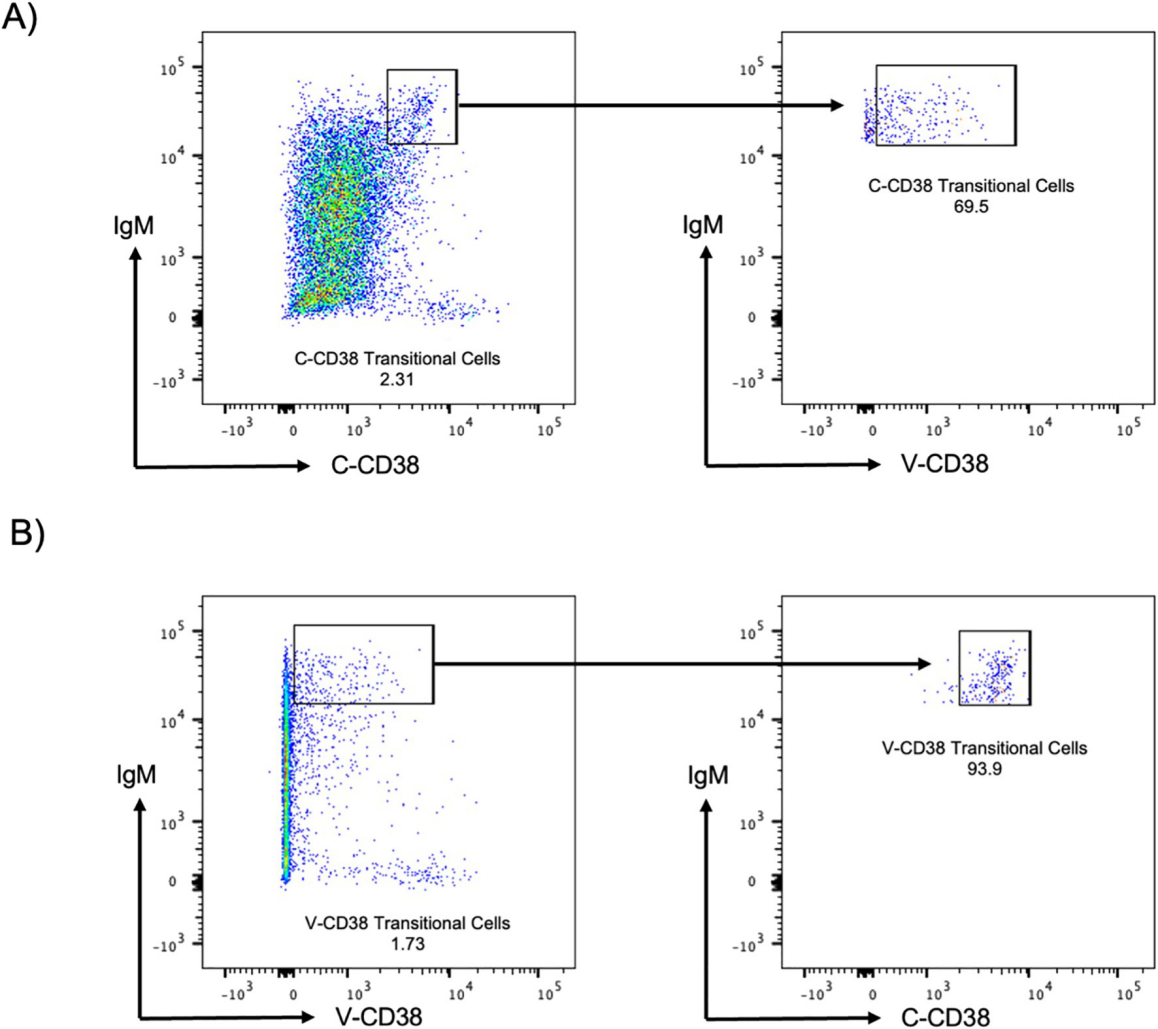
C-CD38 and V-CD38 binding to transitional B cells in peripheral blood. **(A)** Transitional B cells were characterized as single, viable, CD3^-^CD19^+^IgM^hi^C-CD38^int^ cells. The arrows point to the C-CD38 Transitional B cells identified by V-CD38. **(B)** Transitional B cells were characterized as single, viable, CD3^-^CD19^+^IgM^hi^V-CD38^lo^ cells. The arrows point to the C-CD38 Transitional B cells identified by C-CD38.

V-CD38 targets an epitope predicted to be located within the enzymatic cleft of multimeric catalytically active CD38, while C-CD38 recognizes an epitope on the extracellular domain of CD38 independent of conformational changes (15). Therefore, V-CD38 was used to further interrogate the expression of CD38 on transitional B cells. Transitional B cells were characterized as CD3^-^CD19+IgM^hi^CD27^-^CD38^+/++^ following a gating strategy previously described (8). Representative figures of the gating strategy from 6 HC, 6 AbD and 6 AI are provided (**Supplementary Figures 5A–C**, respectively). In contrast to the 1.76% ± 2.83% transitional B cells recognized by C-CD38, significantly (p<0.001) lower frequencies (1.08% ± 2.30%) were identified by V-CD38 (**Figure 4A**), albeit with a strong correlation (r=0.95, p<0.001) between the two antibodies (**Figure 4B**). Across participants, only 61.3% of the C-CD38+ transitional B cells expressed detectable, active multimeric CD38. As seen in the representative **Figure 5A**, only a subset of C-CD38 transitional B cells were recognized by V-CD38 while almost all V-CD38 transitional B cells are recognized by C-CD38 (**Figure 5B**).

**Figure 6:**
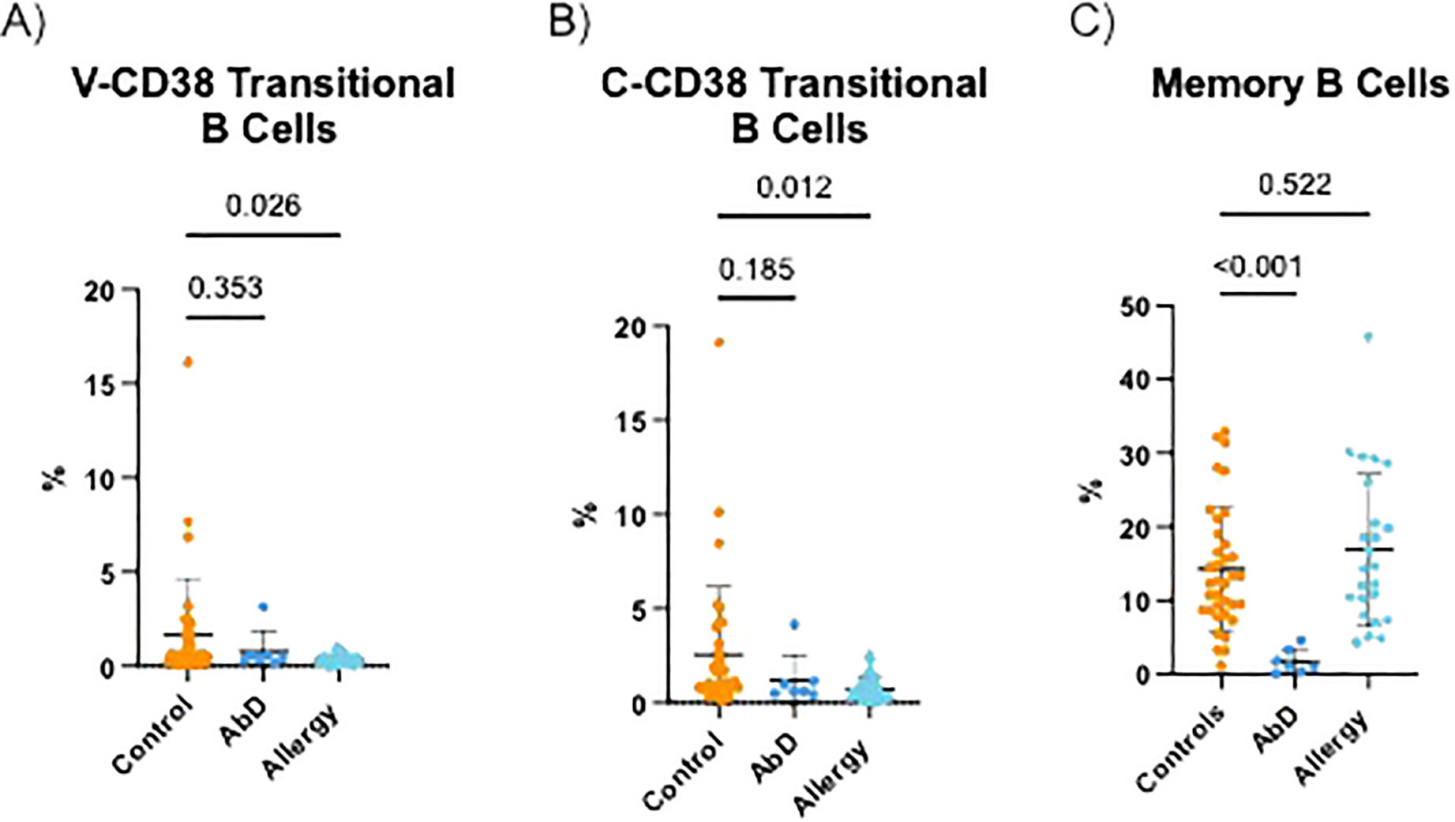
Transitional and memory B cells in the peripheral blood of patients with antibody deficiencies, allergy, and healthy controls. Percentages of CD3^-^CD19^+^CD27^-^IgM^hi^V-CD38^int^ transitional B cells **(A)** CD3^-^CD19^+^CD27^-^IgM^hi^C-CD38^int^ transitional B cells **(B)** and CD3^-^CD19^+^CD27^+^IgD^-^ memory B cells **(C)** in the peripheral blood of HC (n=36), AbD (n=7) and AI (n=24).

It was previously reported that AI have reduced transitional B cells (25). Indeed, as demonstrated in **Figure 6A**, AI had significantly (p=0.025) lower frequencies of V-CD38+ transitional B cells (0.32% ± 0.27%) compared to HC (1.64% ± 3.01%). AbD patients also had lower frequencies of V-CD38 transitional B cells (0.80% ± 1.01%) compared to HC, albeit this difference did not reach statistical significance. AI also had significantly (p=0.012) reduced C-CD38 transitional cells (0.75%±0.67%) compared to HC (2.55% ± 3.62%) (**Figure 6B**). Moreover, among the groups, while 64.4% of HC and 66.1% of AbD patients’ C-CD38 positive transitional B cells were recognized by V-CD38, only 43.4% of AI C-CD38 transitional B cells were detected by V-CD38. The decreased percentages of transitional B cells recognized by C-CD38 or V-CD38 were not due to a decrease in CD3^-^CD19^+^CD27^+^IgD^-^ memory B cells, as the percentages of memory B cells were only reduced significantly (p<0.001) in patients with AbD, but not in AI (**Figure 6C**).

## 4 Discussion

The roles of CD38 in human disease, particularly immune disorders, have been the focus of *extensive research* for more than four decades (2). The availability of animal models and genetic manipulations together with pharmacological and antibody-based agonists and inhibitors have provided important tools for these studies, which are expanded here by demonstrating the potential use of a VLRB-based anti-CD38 monoclonal antibody.

VLRB-MM3 was previously shown to detect plasmablasts in the peripheral blood of healthy individuals 7 days after receiving the influenza vaccine (15). Here we expand this observation and show that V-CD38 plasmablasts are present in PBMC from HC regardless of vaccination status. We also demonstrate that the frequencies of V-CD38 detected plasmablasts are similar to those expected from studies identifying plasmablasts by other methodologies (10, 24, 26, 27). Moreover, while many protocols using C-CD38 define plasmablasts as those expressing “high” levels of CD38, employing V-CD38 enabled gating on all CD38-expressing cells, thereby reducing potential ambiguity. This is particularly advantageous when analyzing samples with reduced percentages of CD38. Indeed, V-CD38 allowed for determining a lower normal value of CD38+ plasmablasts and an easier distinction between HC and patients with AbD. Interestingly, all patients with AbD had reduced V-CD38 plasmablasts, regardless of the cause of the AbD. Hence, although the number of samples assessed here was relatively small, the data presented here suggests that V-CD38 might be beneficial for the analysis of Abd caused by a broad range of defects. Moreover, the consistency and reproducibility of our results suggest that V-CD38 might be advantageous when measuring CD38 expression compared to some of the currently available anti-CD38 antibodies, which will need to be assessed in larger, prospective studies.

Detecting virtually all C-CD38+ plasmablasts by V-CD38, which targets multimeric enzymatically active CD38, suggests that plasmablasts express an enzymatically active CD38. In contrast, only some of the C-CD38+ transitional B cells were recognized by V-CD38, indicating potential heterogeneity of this cell population regarding CD38 enzymatic activity targeting energy metabolism. Thus far, the role of CD38 on transitional B cells has not been well elucidated. The expression of enzymatically active CD38 may align with transitional cell developmental stages (T1, T2, and T3), which are currently poorly understood (28). Hence, our data suggest that V-CD38 might enable further interrogation of transitional B cell classification and maturation.

While AI have plasmablasts that are skewed to the production of IgE responses, it has been reported that they have normal proportions of B cell subsets in their PBMC (29, 30). Indeed, we did not detect significant differences in the percentages of memory B cells and plasmablasts between the AI and healthy control cohort. Instead, we found that transitional B cells, detected by either C-CD38 or V-CD38, were significantly lower in AI, compared to HC. Evidence has been presented that Al patients are deficient in IL-10-producing transitional cells, which act as regulatory B cells, yet it is unclear whether this deficiency would cause a universal decrease in circulating transitional cells (25). It may be possible that the lower transitional B cell populations described here are due to the loss of these regulatory transitional B cells. Alternatively, our observation may reflect the increased age of the AI cohort, as transitional B cell proportions decrease with age (31).

As shown here, V-CD38 can sensitively and specifically detect circulating plasmablasts in diverse AbD, demonstrating its potential to diagnose patients affected by this condition, which is important as some patients present exclusively with defects in the formation of plasmablasts. For example, variants in SEC61A1, IRF2BP2, and TNFSF13 result in plasma cell deficiency and reduced antibody production. Patients with these defects present with hypogammaglobulinemia with normal B cell numbers and circulating memory B cell proportions but with diminished counts of CD27^hi^CD38^hi^ plasmablasts (32–35). Therefore, immunophenotyping with V-CD38 could be very beneficial for their identification. In addition to diagnosing AbD, V-CD38 could be advantageous in targeting CD38 in plasma cell-implicated disorders. Indeed, V-CD38 in CAR T cell therapy might be used to target malignant plasma cells in patients with multiple myeloma. In preclinical studies, CAR-T cells expressing V-CD38 were shown to home and retain at the tumor site in a MISTRG6 xenograft mouse myeloma model (36).

Our study has several limitations. Although some investigators have used the percentages of B cell subpopulations to determine the immune status (37, 38), many others, including the Euroflow guidelines rely on absolute cell counts for the diagnosis of immunodeficiency (5, 10, 26, 27). We intended to demonstrate the potential of V-CD38, therefore here the data were presented as percentages. Future studies, with larger and more diverse cohorts, will be needed to determine the absolute number of V-CD38+ cells indicative of immunodeficiency. These studies will also help establish the optimal cut-off values for V-CD38, as a specificity of 50% is questionable for a biomarker.

In conclusion, our study highlights the potential of VLRB-derived antibodies in clinical use and biomedical research. The limited data presented here suggest that V-CD38, together with other markers and parameters, can help characterize B cells in patients with diverse immune abnormalities and better understand the development of B lineage cells.

## Data Availability

The raw data supporting the conclusions of this article will be made available by the authors, without undue reservation.
