# Supplementary material for: Characterizing CD38 expression in terminally differentiated B cells using variable lymphocyte receptor B tetramers
Source: Front Immunol. 2024 Oct 30;15:1451232. doi: 10.3389/fimmu.2024.1451232 (PMC11579616; doi:10.3389/fimmu.2024.1451232)
Supplement: Supplementary file 1 [file DataSheet1.docx]

Characterizing CD38 Expression in Terminally Differentiated B cells using Variable Lymphocyte Receptor B Tetramers

Arundhati G Nair^1,2^, Matilde Leon-Ponte^1^, Vy HD Kim^3^, Gordon Sussman^4^, Götz R.A. Ehrhardt^5^, Eyal Grunebaum^1,2,3,5^

^1^Developmental and Stem Cell Biology Program, Hospital for Sick Children, Toronto, ON, Canada

^2^Institute of Medical Sciences, University of Toronto, Toronto, ON, Canada

^3^Division of Immunology and Allergy, Department of Pediatrics, The Hospital for Sick Children, Toronto, ON, Canada

^4^Division of Clinical Immunology and Allergy, Department of Medicine, University of Toronto, Toronto, ON, Canada

^5^Department of Immunology, University of Toronto, Toronto, ON, Canada

Abstract

Introduction: CD38 is an ectoenzyme receptor found on hematopoietic cells and its expression is used in the flow cytometric analysis of sub-populations of circulating B cells among peripheral blood mononuclear cells (PBMC) to aid in diagnosing patients with different antibody production defects (AbD). Monoclonal antibodies derived from the sea lamprey Variable Lymphocyte Receptor B (VLRB) are emerging as an alternative to conventional mammalian antibodies. We hypothesized that VLRB MM3 (V-CD38) which specifically recognizes CD38 in a manner correlating with its enzymatic activity could identify terminally differentiated B cells in human PBMC. Here we investigate the ability of V-CD38 as a tool to diagnose patients with diverse immune abnormalities including AbD.

Methods: The expression of CD38 on CD3^-^CD19^+^CD27^+^ plasmablasts and CD3^-^CD19^+^IgM^hi^CD27^-^ transitional B cells in PBMC were analyzed by flow cytometry using V-CD38 and compared with a commercial conventional antibody to CD38 (C-CD38).

Results: A highly significant correlation (p<0.001, r=0.99) between the percentages of plasmablasts recognized by V-CD38 and C-CD38 was observed among 36 healthy controls (HC), 7 patients with AbD and 24 allergic individuals (AI). The use of V-CD38 enabled improved gating of the CD38 expressing cells (CD38+), aiding in the observation that patients with AbD had significantly lower (p=0.002) CD38+ plasmablasts (0.13%±0.13%) than HC (0.52%±0.57%). Only 61.3% of the transitional B cells detected by C-CD38 were also recognized by V-CD38 (r=0.95, p<0.001) among the 67 participants. AI had significantly reduced V-CD38 and C-CD38 transitional cells compared to HC (p=0.026 and p=0.012, respectively).

Conclusions: V-CD38 is a novel reagent that can assess B cells in human PBMC.

Supplemental Figures and Tables

**Supplemental Figures**

Figure 1. Binding of the biotinylated VLRB MM3, VLRB B7 and HB-7 to Daudi cells.


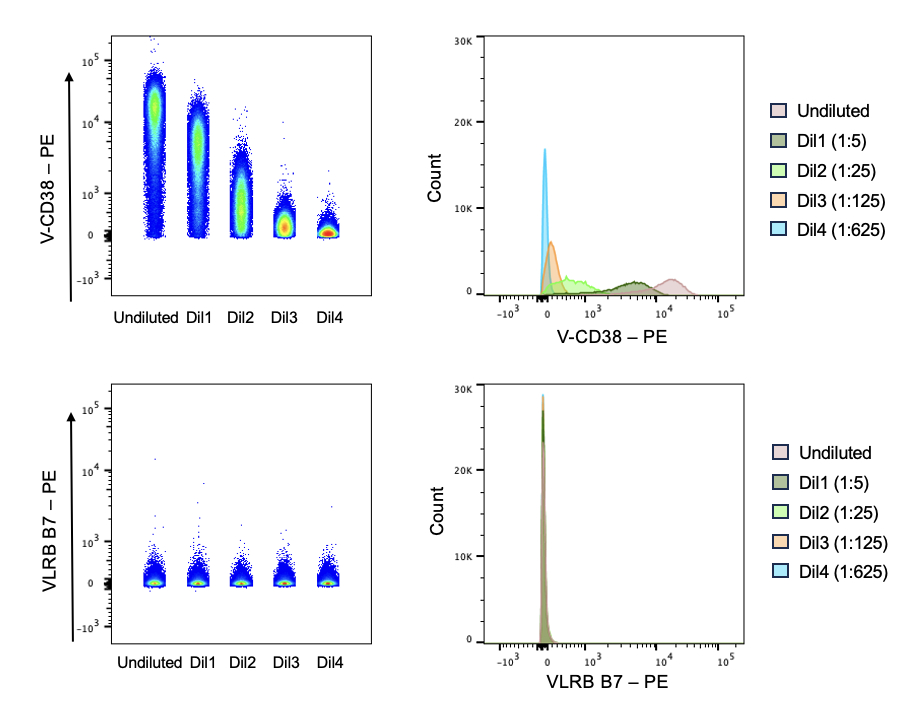


A)

B)

A) V-CD38 binding to Daudi cells. The left figure demonstrates the binding and titration of biotinylated VLRB-MM3 (V-CD38) at dilutions 1:5 (Dil1), 1:25 (Dil2), 1:125 (Dil3), 1:625 (Dil4), and undiluted. The right figure is a histogram of the fluorescence intensities for each V-CD38 dilution. The figures are representative of 3 independent repeats.

B) VLRB B7 binding to Daudi cells. The left figure demonstrates the binding and titration of biotinylated VLRB-B7 (against the SARS-CoV-2 spike protein) at dilutions 1:5 (Dil1), 1:25 (Dil2), 1:125 (Dil3), 1:625 (Dil4), and undiluted. The right figure is a histogram of the fluorescence intensities for each VLRB B7 dilution. The figures are representative of 3 independent repeats.


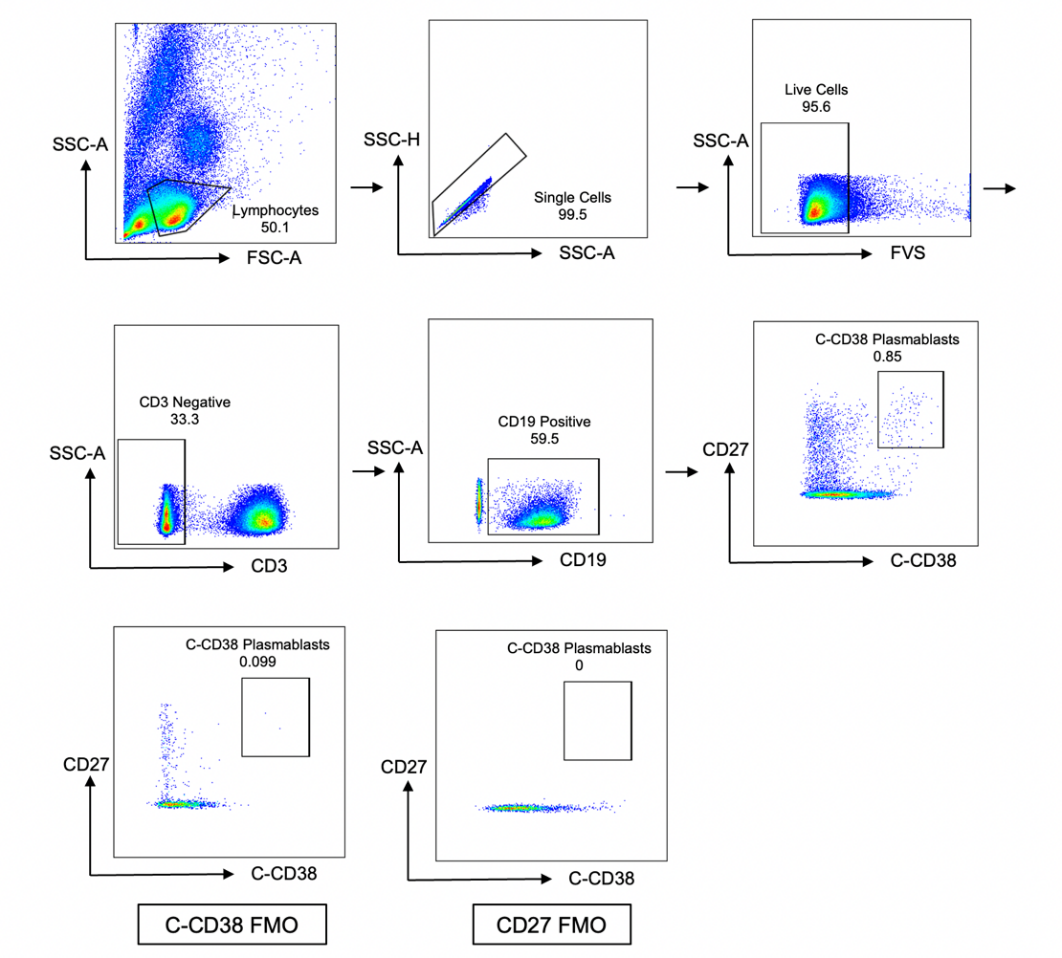

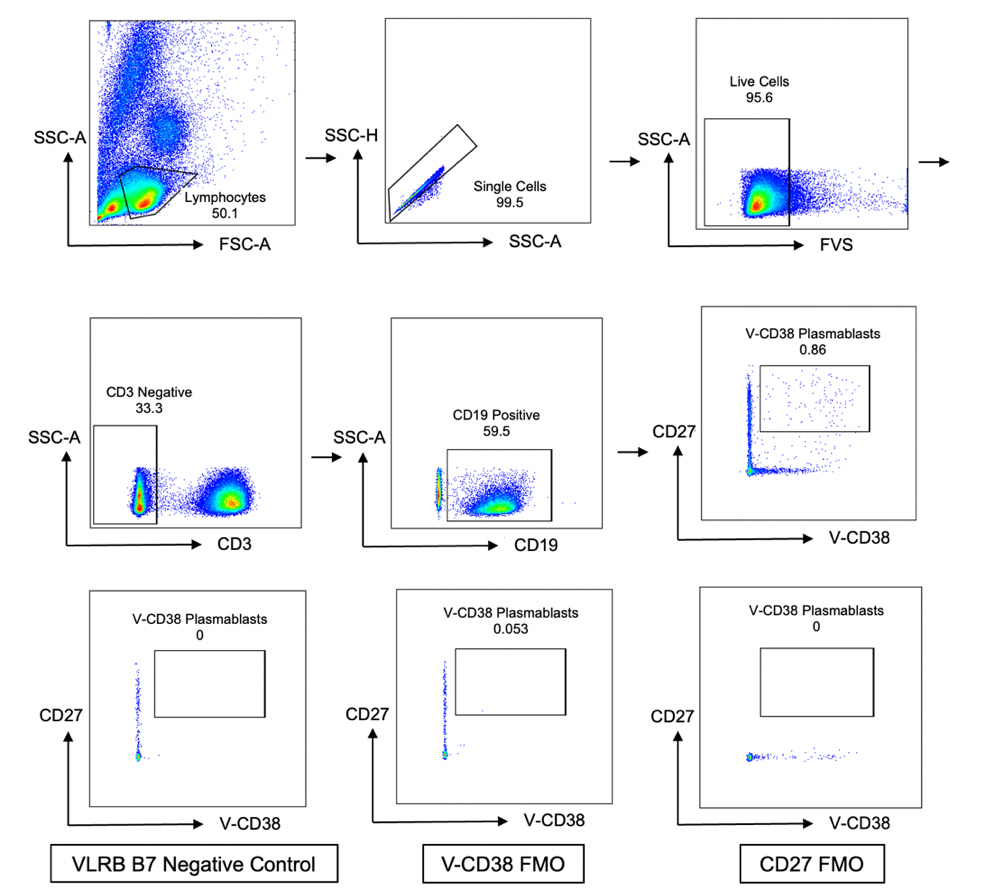
Figure 2. Gating strategy to identify plasmablasts using V-CD38 and C-CD38 in peripheral blood.

B)

A)

1. Gating strategy to identify plasmablasts with V-CD38. Plasmablasts from peripheral blood mononuclear cells were identified as single, live, CD3^-^CD19^+^ cells, with high expression of CD27 and binding of V-CD38 (CD3^-^CD19^+^CD27^hi^V-CD38^+^). Staining with VLRB B7, used as a negative control, and FMOs for V-CD38 and CD27 are also provided. The figure is representative of the analysis performed from 3 healthy controls.
2. Gating strategy to identify plasmablasts with C-CD38. Plasmablasts from peripheral blood mononuclear cells were characterized as single, live, CD3^-^CD19^+^ cells, with high expression of CD27 and binding by C-CD38 (CD3^-^CD19^+^CD27^hi^C-CD38^+^). FMOs for C-CD38 and CD27 are also provided. The figure is representative of the analysis performed from 3 healthy controls.


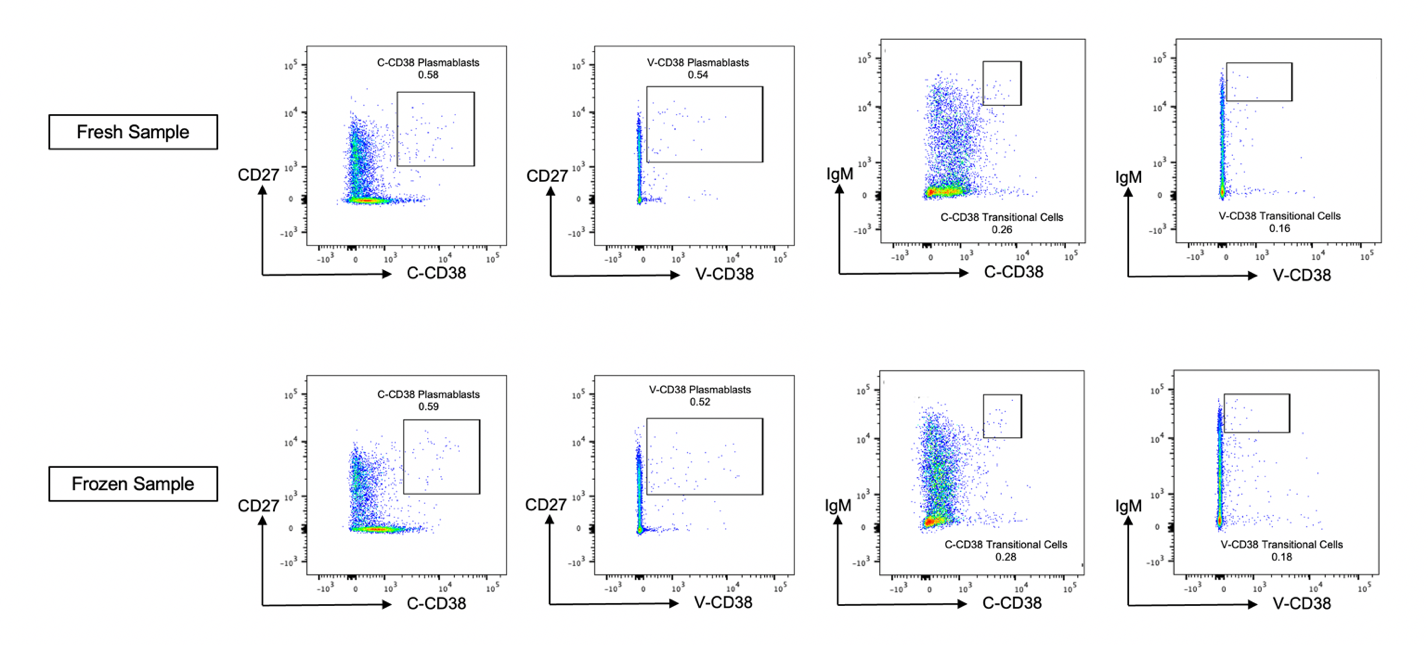
Figure 3: CD38 expression on fresh and thawed peripheral blood mononuclear cells.

A)

B)

1. Representative example of fresh and thawed single live CD3^-^CD19^+^ B cells from a healthy control’s peripheral blood mononuclear cells, identified with C-CD38^+^ or V-CD38^+^.
2. The percentages of fresh and thawed single live CD3^-^CD19^+^ B cells from peripheral blood mononuclear cells of 3 healthy controls, identified with C-CD38^+^ or V-CD38^+^.

Figure 4: CD38 expression on IgG-producing plasmablasts

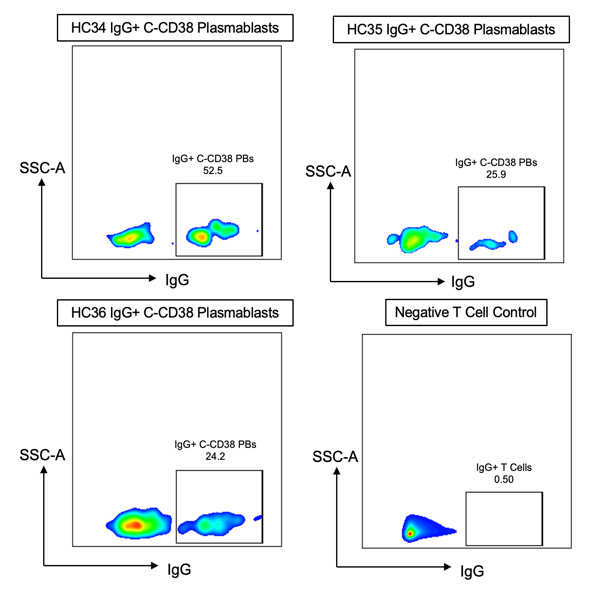


B)

A)


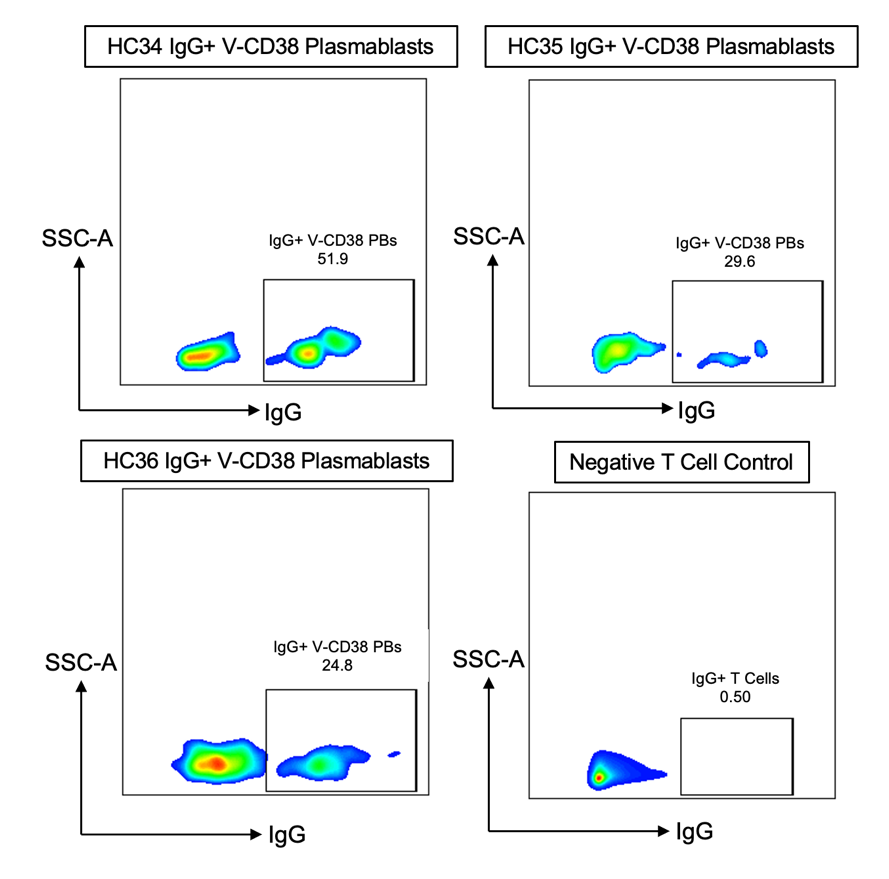


C)

1. V-CD38 recognizes IgG-producing plasmablasts. IgG-producing CD3^-^CD19^+^CD27^hi^V-CD38^+^ (in the rectangle) were identified in 3 healthy controls (HC) using a density plot to differentiate the negative and positive populations. CD3^+^ T cells were used as a negative control for gating.
2. C-CD38 recognizes IgG-producing plasmablasts. IgG-producing CD3^-^CD19^+^CD27^hi^C-CD38^+^ (in the rectangle) were identified in 3 healthy controls (HC) using a density plot to differentiate the negative and positive populations. CD3^+^ T cells were used as a negative control for gating.
3. The percentages of IgG-producing plasmablasts recognized by V-CD38 and C-CD38. The percentages of IgG-producing V-CD38^+^ or C-CD38^+^ plasmablasts in PBMC from 3 healthy controls.


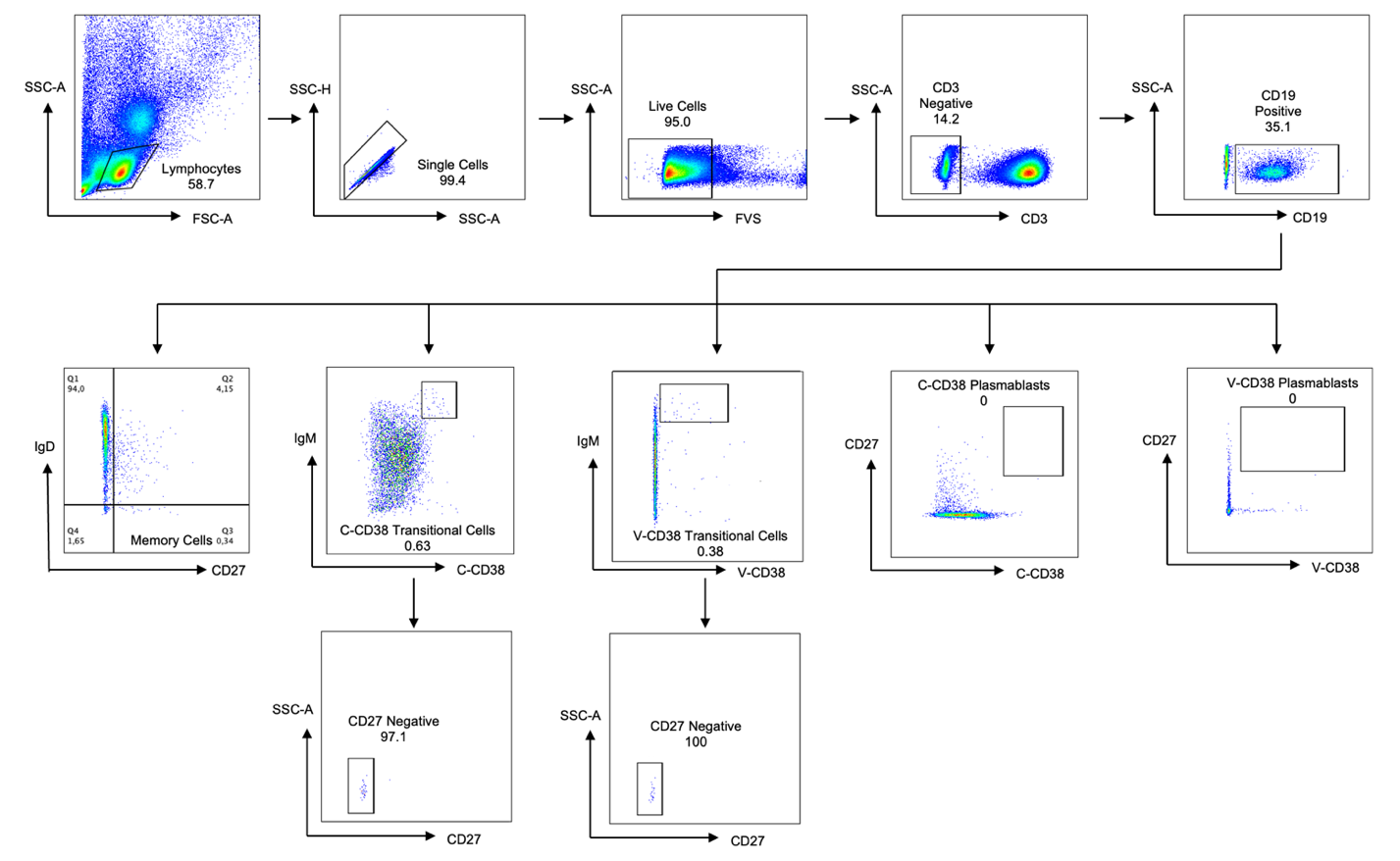

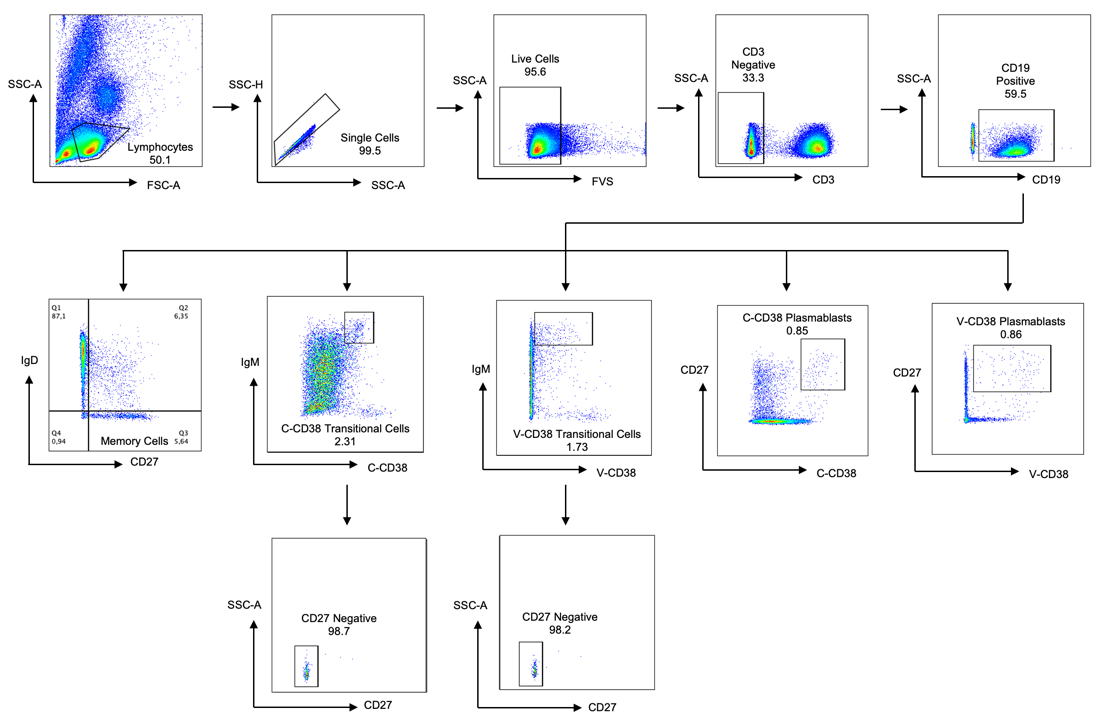
Figure 5: Gating strategy to identify transitional and memory B cells in the peripheral blood.

B)

A)

C)


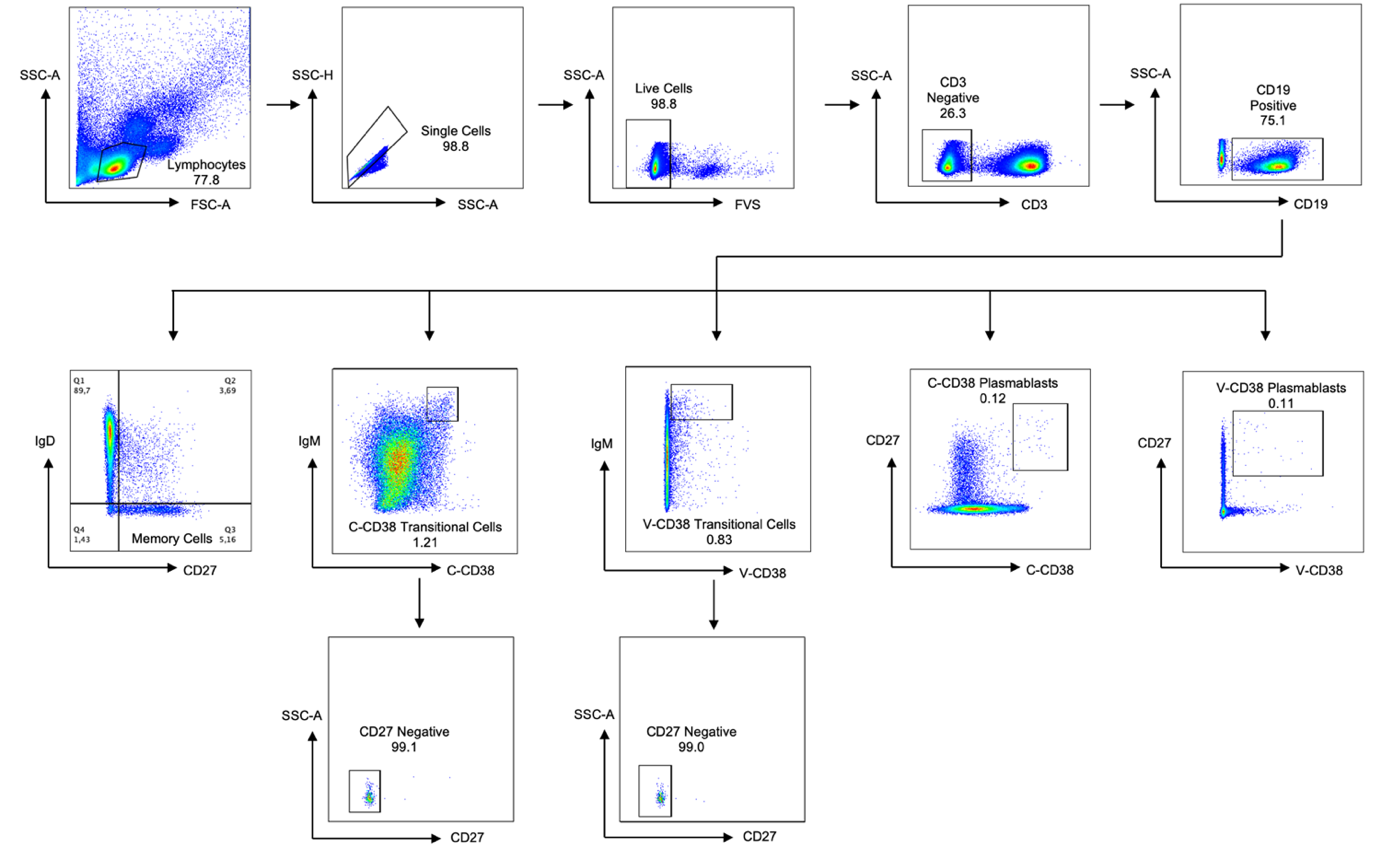


Single, viable, CD3^-^CD19^+^ peripheral blood mononuclear cells were characterized as CD27^+^IgD^-^ memory cells, IgM^hi^C-CD38^int^CD27^-^ or IgM^hi^V-CD38^lo^CD27^-^ transitional cells and C-CD38^hi^CD27^+^ or V-CD38^hi^CD27^+^ plasmablasts.

A). A representative figure of memory B cells, transitional cells and plasmablasts from healthy controls. N=6.

B). A representative figure of memory B cells, transitional cells and plasmablasts from patients with antibody deficiencies. N=6.

C). A representative figure of memory B cells, transitional cells and plasmablasts from allergic individuals. N=6.

**Supplemental Tables**

Table 1. Details of patients with abnormal antibody production

| - Patient # | - Age - (y) | - Sex | - IgG g/l | - IgM - g/l | - IgA - g/l | - Diagnosis (gene defect, if known) | - CD19^+^ cells/ul | - Transitional % | - Switched Memory - % | - Plasmablasts - % |
| --- | --- | --- | --- | --- | --- | --- | --- | --- | --- | --- |
| - 1 | - 17 | - M | - IG* | - **0.3** | - **<0.1** | - Hypogamma post HSCT (LRBA) | - 354 | - 0.8 | - **0** | - **0.4** |
| - 2 | - 17 | - M | - **4.5** | - 0.5 | - 0.6 | - Hypogamma recurrent infections | - 474 | - **0.1** | - **5.1** | - **0.1** |
| - 3 | - 4 | - M | - IG* | - **<0.1** | - **<0.1** | - Agamma (BTK) | - **0** | - **0** | - **0** | - **0** |
| - 4 | - 12 | - M | - IG* | - **<0.1** | - **<0.1** | - Agamma (BTK) | - **0** | - **0** | - **0** | - **0** |
| - 5 | - 16 | - M | - IG* | - 0.5 | - **<0.1** | - Hypogamma, autoimmunity | - 178 | - **0.1** | - **0.4** | - **0** |
| - 6 | - 19 | - M | - 9.4 | - 0.7 | - 0.9 | - HyperIgE synd, recurrent infections, abnormal Ab (STAT3) | - **302** | - **4.2** | - **1.9** | - **0.2** |
| - 7 | - 19 | - M | - **3.8** | - 0.5 | - **0.3** | - Hypogamma, autoimmunity | - 177 | - **1** | - **2** | - **0.1** |
| - 8 | - 26 | - F | - IG* | - 0.4 | - **0.1** | - Hypogamma (ATm) | - **19** | - ND**** | - ND**** | - ND**** |
| - 9 | - 17 | - M | - **6.3** | - 0.9 | - 0.6 | - Hypogamma, (SON) | - 340 | - 2.2 | - 3.0 | - **0.2** |
| - 10 | - 11 | - M | - **4.7** | - 0.4 | - **0.2** | - Hypogamma, autoimmunity | - 443 | - 2.2 | - 2.3 | - 0.3 |

*IG- Receiving immunoglobulin replacement, Data in **bold** indicates abnormal values for age.

Abbreviations: Ab- antibody production, Agamma- agammaglobulinemia, ATm- Ataxia-Telangiectasia mutated, BTK- Bruton’s Tyrosine Kinase, HSCT- hematopoietic stem cell transplantation, Hypogamma- hypogammaglobulinemia, LRBA- lipopolysaccharide-responsive and beige-like anchor, ND- not done, SON- SON haploinsufficiency causing ZTTK syndrome, STAT3-signal transducer and activator of transcription 3.

Table 2. Sensitivity and specificity of V-CD38 and C-CD38 in identifying decreased (<0.35%) plasmablasts in patients with abnormal antibody production.

|  | V-CD38 | C-CD38 |
| --- | --- | --- |
| Sensitivity | 100% | 86% |
| Specificity | 50% | 44% |
